# Myoclonus Secondary to Amantadine: Case Report and Literature Review

**DOI:** 10.3390/clinpract13040075

**Published:** 2023-07-20

**Authors:** Jamir Pitton Rissardo, Ana Letícia Fornari Caprara

**Affiliations:** Medicine Department, Federal University of Santa Maria, Santa Maria 97105-900, Brazil; ana.leticia.fornari@gmail.com

**Keywords:** Parkinson’s disease, myoclonus, movement disorder, amantadine, 1-adamantylamine

## Abstract

The usual adverse events of amantadine are dizziness, dry mouth, and peripheral edema. Postmarketing experience has revealed abnormal movements such as tremors, involuntary muscle contractions, and gait abnormalities. Herein, we report a case of an elderly male who presented with generalized twitching associated with amantadine. A 64-year-old male presenting with jerking movements within one day of onset was admitted. Sudden and involuntary distal lower and upper limb muscle twitching was observed. The subject presented subsequent brief movements when attempting to stand or hold arms antigravity. He was diagnosed with Parkinson’s disease three years ago. Eight days before the presentation to the emergency department, he consulted with his primary care physician, who prescribed amantadine to improve his motor symptoms. On the seventh day, he developed brisk abnormal movements. Laboratory exams, neuroimaging, and electroencephalogram were unremarkable. Amantadine was discontinued. After three days, the patient reported that his jerking movements had fully recovered. To the authors’ knowledge, 22 individuals with amantadine-associated myoclonus had already been reported in the literature. The pathophysiology of amantadine-induced myoclonus is probably related to serotoninergic pathways. Myoclonus secondary to amantadine was slightly more common in men. The population affected was elderly, with a mean and median age of 67.7 and 64 years.

## 1. Introduction

Amantadine hydrochloride was developed as an antiviral medication. One of the pioneer amantadine clinical trials showed improvement in the influenza virus infection course and motor symptoms of patients affected by Parkinson’s disease. In 1973, the Food and Drug Administration approved amantadine for treating Parkinson’s disease [1].

The most common adverse events of amantadine are dizziness, dry mouth, peripheral edema, and livedo reticularis. Postmarketing experience has revealed that some patients could develop abnormal movements such as tremors, involuntary muscle contractions, and gait abnormalities [2,3]. To the authors’ knowledge, 22 individuals of amantadine-associated myoclonus have already been reported in the literature [2,4,5,6,7,8,9,10,11,12,13,14,15,16,17,18].

Herein, we present a case of an elderly male who presented with generalized twitching movements, probably secondary to amantadine. Moreover, we provide a table with all the reported cases and present a figure describing the therapeutic range based on amantadine reports and clinical trials.

## 2. Case Report

A 64-year-old male presenting with jerking movements within one day of onset was admitted to our emergency department. He reported that the abnormal involuntary movements became worse throughout the last day. His vital signs, such as temperature, heart rate, respiratory rate, and blood pressure, were within normal limits.

On neurological examination, sudden and involuntary distal lower and upper limb muscle twitching was observed. Additionally, the subject presented subsequent brief movements when attempting to stand or hold arms against gravity. Bradykinesia, tremor, and postural instability associated with shuffling gait were noted. The muscle mass and strength were normal (Grade 5—Medical Research Council). The assessment of cranial nerves was unremarkable. Deep tendon reflexes were normal and active. His neurological family history was unremarkable. Additionally, he had no history of known drug allergies or adverse drug reactions.

He was diagnosed with Parkinson’s (Hoehn and Yahr stage II) three years ago. In the last month, the individual reported having more common off-periods with difficulty walking. He only used 100 mg levodopa + 25 mg benserazide tablet thrice daily. Eight days before the presentation to the emergency department, he consulted with his primary care physician, who added amantadine to improve his motor symptoms. Amantadine hydrochloride 100 mg tablet once a day for three days was started. After three days, he increased the amantadine dosage to one tablet twice daily. On the seventh day, he began with brisk abnormal movements.

Laboratory exams were within normal limits, including serum creatinine levels. A cranial computed tomography scan was normal. A brain magnetic resonance (1.5 Tesla) was normal without changes after contrast material. Cerebrospinal fluid analysis showed 60 mg/dL of glucose (98 mg/dL plasma glucose), 30 mg/dL protein, 0 leukocytes, and 0 red blood cells. An electroencephalogram was normal without background seizure activity, slowing, or suppressions.

On the second admission day, it was observed that the symptoms worsened approximately two hours after the amantadine administration (Video S1). It was hypothesized that his myoclonus was probably associated with amantadine. Clonazepam and hydration were started. Amantadine was discontinued. After three days, the patient reported that his jerking movements had fully recovered. In the long-term follow-up of one year, the patient did not have a recurrence of the myoclonus. He continued with his baseline Parkinson’s symptoms and was administered 100 mg levodopa + 25 mg benserazide, one tablet, five times daily.

## 3. Discussion

**Figure 1:**
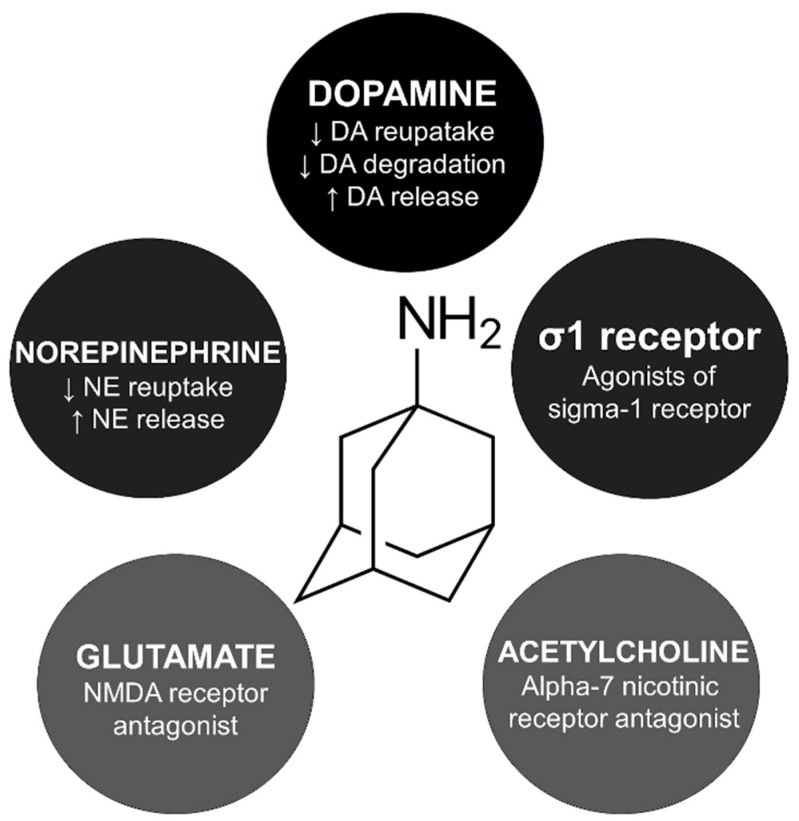
Mechanism of action and skeletal formula of amantadine. DA, dopamine; NE, norepinephrine; NMDA, N-methyl-D-aspartate.

The exact mechanism of amantadine for managing parkinsonism and drug-induced extrapyramidal reactions is unknown. However, five main indirect pathways have been studied to explain amantadine’s involvement in the cortico-striato-pallido-thalamo-cortical loop (Figure 1).

Amantadine is believed to activate presynaptic dopamine receptor D2 reducing the dopamine transporter related to dopamine reuptake [19]. In animal studies, amantadine inhibited monoamine oxidase B, decreasing dopamine degradation [20]. Interestingly, amantadine is an agonist of the σ1 receptors, which is probably related to the psychostimulant-like effects of this compound [21]. Amantadine blocks alpha-7 nicotinic receptors, which explains its anticholinergic side effects like xerostomia, urinary retention, and constipation [22].

Amantadine may influence serotoninergic pathways, increasing serotonin availability. In rat models, this drug increases serotonin release and inhibits serotonin reuptake in presynaptic neurons [23,24]. One of the hypotheses for the pathophysiology of myoclonus is related to serotonin augmentation in the cerebellar output [25]. Therefore, myoclonus secondary to amantadine could be related to a serotonergic mechanism. A similar explanation has been hypothesized for other drug-induced myoclonus, such as lithium and fluoroquinolones [26,27]. Notably, these drugs share case presentation similarities regarding myoclonus and progressive cognitive impairment, known by some authors as Creutzfeldt–Jakob-like syndrome.

We searched six databases to locate the studies on amantadine and myoclonus published from 1980 to June 2022 in electronic form. Excerpta Medica (Embase), Google Scholar, Latin American & Caribbean Health Sciences Literature (Lilacs), Medline, Scientific Electronic Library Online (Scielo), and Science Direct were searched. Search terms were “myoclonus, movement disorder”. These terms were combined with “amantadine, 1-adamantylamine” (Table S1). Publications in English were included in the search (Table 1) [2,4,5,6,7,8,9,10,11,12,13,14,15,16,17,18].

The most common MCL presentation was multifocal jerks in the limbs, but focal jerks were also observed. In this context, some cases that described cranial myoclonus, also known as branchial or vocal myoclonus, involving facial muscles were misdiagnosed as stuttering [4,10]. Notably, amantadine is a type of NMDA antagonist, like ketamine and phencyclidine, which are well known to cause a head-twitch response in rat models [28]. Therefore, the association of myoclonus with amantadine therapy is expected. Notably, amantadine was prescribed for many different types of disorder (e.g., Parkinson’s disease, progressive supranuclear palsy, disorders of consciousness, and depression), so differences from a pathophysiological point of view on the role of amantadine in these very different conditions is possible.

Amantadine-induced myoclonus was slightly more common in men (12/23). The population affected was the elderly, with a mean and median age of 67.7 (SD: 9.8) and 64 years (age range: 53–87). It is worth mentioning that the studied population involved patients with Parkinson’s disease, which can explain the age of the people affected [29].

Most of the cases were not reported by movement disorder specialists, which could have led to possible misdiagnosis of the abnormal movement in some reports. In only 43% (10/23) of the cases, electrodiagnostic studies were performed. The description of supporting studies is essential for defining myoclonus sources [30]. The most frequent source of myoclonus was cortical, but some authors reported a subcortical origin.

**Figure 2:**
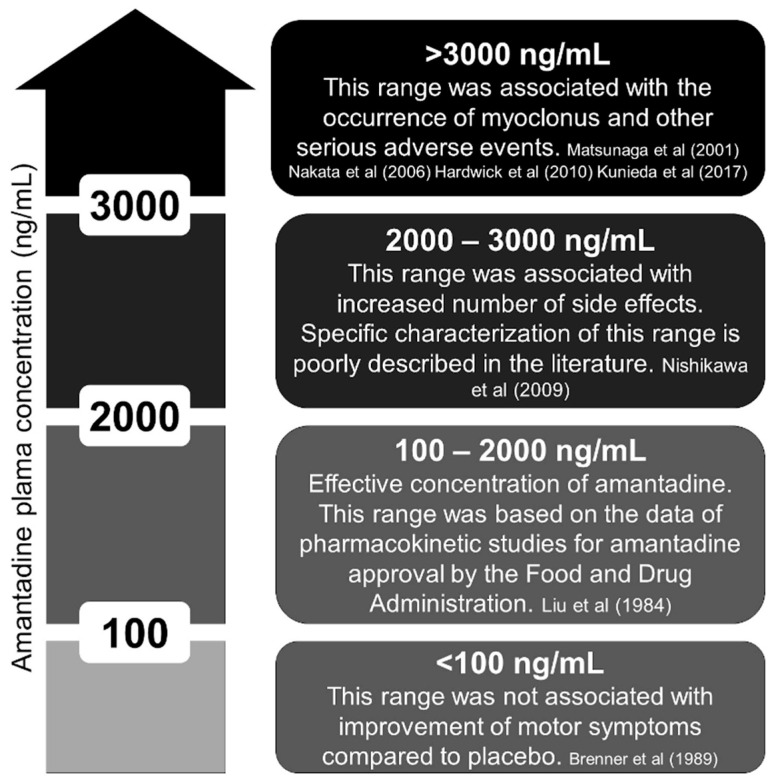
Plasma concentration of amantadine (ng/mL). Ineffective, effective, unknown, and toxic ranges [5,6,9,11,16,33,34].

In pharmacokinetic studies, the plasma concentration of amantadine ranged from 100 to 2000 ng/mL [31]. In elderly individuals, 1000 to 2000 ng/mL is considered dangerous by some authors due to a higher incidence of side effects such as hallucinations and delirium [31,32]. We reviewed the literature and provide a figure about the amantadine concentration (Figure 2) [5,6,9,11,16,33,34]. Interestingly, all the cases that reported myoclonus had amantadine concentrations above 3000 ng/mL [9]. Despite prevailing renal elimination, the metabolism of amantadine is not yet fully clarified because 5–15% of an oral dose is apparently acetylated and acetylator phenotype might influence toxicity [35].

The availability and costs regarding the measurement of serum amantadine levels are still a limitation to a specific approach to the adverse events associated with this medication. In this context, developing extended-release formulations with late peak plasma concentration and a longer half-life may be associated with increased side effects. Thus, assessing drug levels for adequately managing parkinsonism will be mandatory. Meanwhile, clinicians should rely on the Cockcroft–Gault formula to estimate creatinine clearance as a risk factor for developing side effects related to amantadine. Noteworthy, levodopa, considered the mainstay of treating Parkinson’s disease, was already associated with myoclonus. However, levodopa-induced myoclonus is a relatively late complaint because most individuals only present this side effect after ten years of levodopa use [36].

Eighty to ninety percent of amantadine is excreted unchanged by glomerular filtration and tubular secretion. In this way, renal dysfunction can cause accumulation of this drug in several organs, such as the lungs and kidneys. Interestingly, the approximate half-life of amantadine is sixteen hours in individuals with normal renal function and eight days in dialytic individuals [31]. Therefore, we analyzed the data of the cases regarding renal impairment in the individuals reported in Table 1. Of the 23 subjects, 12 had at least mildly decreased renal function. However, some authors did not describe creatinine levels or creatinine clearance.

Dames et al. reported a case of a patient falling several times a day for years. The authors reported a possible association between falling and amantadine therapy. In this context, they observed that the patient had generalized myoclonus contributing to his imbalance. The amantadine clinical trials revealed an increased dose-dependent percentage of falls in patients with Parkinson’s disease [37,38]. GOCOVRI’s trials for levodopa-induced dyskinesia demonstrated a higher incidence of falls with amantadine. This finding was mainly observed in patients over 65 years old [39].

## 4. Conclusions

Myoclonus secondary to amantadine has rarely been reported in the literature. The pathophysiology of this association is probably related to serotoninergic pathways. Clinicians should consider amantadine-induced myoclonus as a cause of falling in Parkinson’s disease patients. Future reports should describe electrodiagnostic studies for a determination of the myoclonus source.

## Figures and Tables

**Table 1:** Clinical reports of myoclonus associated with amantadine.

Reference (Year)	Age/Sex	AMT Dosing (mg/Daily) and Indication	MCL Presentation	KF	MCL Onset ^a^	Management	MCL Recovery ^b^	EEG ^c^	F/U	Considerations
Chevalier et al. (1980) [2]	64, M	NA; PD	Generalized MCL.	N	NA	AMT withdrawal	NA	NA	NA	First report of AMT-induced MCL. Diuretics increased the intoxication by AMT.
Pfeiffer et al. (1996) [4]	64, F	200; PD	Focal (cranial) MCL.	N	NA	AMT withdrawal. Clonazepam was attempted.	NA	NA	NA	First report of vocal (cranial) MCL. Misdiagnosed with stuttering. Videotape.
Matsunaga et al. (2001) [5]	87, F	100; NA	Generalized (multifocal) MCL. Cortical MCL.	Y	30 days	AMT withdrawal	14 days	Abnormal	CR	Plasma AMT concentration.
	78, F	200; PD	Generalized (multifocal) MCL. Cortical MCL.	N	90 days	AMT withdrawal	8 days	Abnormal	CR	Dose-dependent MCL. AMT-dose increase was associated with a rise in MCL frequency.
	79, F	200; PD	Generalized (multifocal) MCL. Cortical MCL.	Y	9 days after worsening of renal function	AMT withdrawal	7 days	Abnormal		Plasma AMT concentration. MCL appeared with worsening renal function.
Nakata et al. (2006) [6]	70, F	150; PD	Generalized MCL.	Y	NA (y)	AMT withdrawal	21 days	Normal	CR	Plasma AMT concentration.
	74, F	200; Depression	Generalized MCL	N	NA (y)	AMT withdrawal	21 days	Normal	CR	Possible serotonin syndrome.
	73, F	300; PD	Generalized MCL.	Y	7 days	AMT withdrawal	NA	Abnormal	No	Possible serotonin syndrome.
Cheng et al. (2008) [7]	78, M	100; PD	Generalized MCL	Y	3 days	AMT withdrawal	12 days	Abnormal	CR	Serotonin syndrome.
Hong et al. (2008) [8]	59, F	200; PD	Generalized MCL	Y	11 days	AMT withdrawal	NA	NA	NA	Possible interaction with pramipexole.
Nishikawa et al. (2009) [9]	62, F	200; PD	Generalized MCL	Y	NA	AMT withdrawal	NA	NA	CR	Plasma AMT concentration.
	55, F	150; PD	Generalized MCL	Y	NA	AMT withdrawal	NA	NA	CR	Plasma AMT concentration.
Gupta et al. (2010) [10]	63, M	300; parkinsonism with postural instability	Focal (cranial) MCL. Resting and action MCL of lower face.	N	NA (several months)	AMT withdrawal	NA	NA	CR	Videotape. Misdiagnosed as stuttering.
Hardwick et al. (2010) [11]	63, M	NA; pruritus	Generalized MCL	Y	NA	AMT withdrawal	56 days	Normal	CR	Plasma AMT concentration.
Yarnall et al. (2012) [12]	74, M	200; PSP	Generalized MCL	N	26 days	AMT withdrawal	5 days	NA	CR	PSP diagnosis supported by abnormal DaTSCAN.
Kawamura et al. (2013) [13]	58, M	NA; PSP	Generalized MCL	N	NA	Clonazepam was attempted	NA	NA	NA	Giant potential was found in somatosensory evoked potential of the median nerve.
Estraneo et al. (2015) [14]	57, F	200; coma state	Focal (cranial) MCL	N	21 days	AMT withdrawal	21 days	Abnormal	NA	Three attempts of AMT rechallenge.
Janssen et al. (2017) [15]	66, M	300; PD with Levodopa-induced dyskinesias	Generalized MCL	N	30 days	AMT withdrawal	14 days	NA	CR	Videotape.
Kunieda et al. (2017) [16]	83, M	150; PD	Generalized MCL.	Y	5 days	AMT withdrawal	29 days	NA	CR	Plasma AMT concentration
	53, M	100; spontaneity	Generalized MCL.	Y	21 days	AMT withdrawal. AMT rechallenge.	NA	NA	CR	AMT rechallenge without symptoms occurrence.
Dames et al. (2020) [17]	55, M	400; PD	Generalized MCL.	Y	NA (y)	AMT withdrawal	7 days	NA	No	Videotape.
Poon et al. (2021) [18]	80, M	PD with Levodopa-induced dyskinesias	Generalized MCL. Asterixis.	N	9 days	AMT withdrawal	3 days	NA	CR	Subcortical MCL.
Present report	64, M	PD	Generalized MCL. Asterixis.	N	7 days	AMT withdrawal	3 days	Normal	CR	Subcortical MCL.

Abbreviations: AMT, amantadine; CR, complete recovery; EEG, electroencephalography; F, female; F/U, follow-up; KF, kidney failure/renal dysfunction; M, male; MCL, myoclonus; N, no; NA, not available/not reported; PD, Parkinson’s disease; PSP, progressive supranuclear palsy; Y, yes; y, years. ^a^ MCL onset: time from AMT starting until the MCL onset. ^b^ MCL recovery: time from AMT withdrawal (management) until MCL recovery. ^c^ Some of the EEG abnormalities were increased predominant background alpha activity and intermittent generalized diffuse slow waves.

## Data Availability

Not applicable.

## Supplementary Materials

The following supporting information can be downloaded at: https://www.mdpi.com/article/10.3390/clinpract13040075/s1, Video S1: Asterixis, Table S1: FreeText and MeSH search terms in the US National Library of Medicine.

## References

[1] Rascol O., Fabbri M., Poewe W. (2021). Amantadine in the treatment of Parkinson’s disease and other movement disorders. Lancet Neurol..

[2] Chevalier J.F., Renier E., Brion S. (1980). Edema and myoclonus in a patient with Parkinson’s disease treated by amantadine. L’encephale.

[3] Marmol S., Feldman M., Singer C., Margolesky J. (2021). Amantadine Revisited: A Contender for Initial Treatment in Parkinson’s Disease?. CNS Drugs.

[4] Pfeiffer R.F. (1996). Amantadine-induced “vocal” myoclonus. Mov. Disord..

[5] Matsunaga K., Uozumi T., Qingrui L., Hashimoto T., Tsuji S. (2001). Amantadine-induced cortical myoclonus. Neurology.

[6] Nakata M., Ito S., Shirai W., Hattori T. (2006). Severe reversible neurological complications following amantadine treatment in three elderly patients with renal insufficiency. Eur. Neurol..

[7] Cheng P.L., Hung S.W., Lin L.W., Chong C.F., Lau C.L. (2008). Amantadine-induced serotonin syndrome in a patient with renal failure. Am. J. Emerg. Med..

[8] Hong C.T., Sun Y., Lu C.J. (2008). Fatal intoxication using amantadine and pramipexole in a uremic patient. Acta Neurol. Taiwan..

[9] Nishikawa N., Nagai M., Moritoyo T., Yabe H., Nomoto M. (2009). Plasma amantadine concentrations in patients with Parkinson’s disease. Park. Relat. Disord..

[10] Gupta A., Lang A.E. (2010). Drug-induced cranial myoclonus. Mov. Disord..

[11] Hardwick A., Devereaux M., Walter B. (2010). A Case of Subacute Encephalopathy, Ataxia and Myoclonus Due to Amantadine Toxicity in Chronic Renal Insufficiency. Mov. Disord..

[12] Yarnall A.J., Burn D.J. (2012). Amantadine-induced myoclonus in a patient with progressive supranuclear palsy. Age Ageing.

[13] Kawamura K., Arii Y., Inui T., Mitsui T. (2013). A case of progressive supranuclear palsy with cortical myoclonus. Tokushima.

[14] Estraneo A., Pascarella A., Moretta P., Loreto V., Trojano L. (2015). Clinical and electroencephalographic on-off effect of amantadine in chronic non-traumatic minimally conscious state. J. Neurol..

[15] Janssen S., Bloem B.R., Warrenburg B.P. (2017). The clinical heterogeneity of drug-induced myoclonus: An illustrated review. J. Neurol..

[16] Kunieda K., Shigematsu T., Fujishima I. (2017). Case Reports Describing Amantadine Intoxication in a Rehabilitation Hospital. Prog. Rehabil. Med..

[17] Dames B., Karl J.A., Metman L.V. (2020). High dose amantadine therapy may cause increased falling in patients with Parkinson’s disease: A case report. Clin. Park. Relat. Disord..

[18] Poon L.H., Lee A.J., Vuong M., Zuzuarregui J.R. (2021). Amantadine Associated Myoclonus: Case Report and Review of the Literature. J. Pharm. Pract..

[19] Raupp-Barcaro I.F.M., Dias I.C.S., Meyer E., Vieira J.C.F., Pereira G.S., Petkowicz A.R., Oliveira R.M.W., Andreatini R. (2021). Involvement of dopamine D(2) and glutamate NMDA receptors in the antidepressant-like effect of amantadine in mice. Behav. Brain Res..

[20] Strömberg U., Svensson T.H. (1971). Further studies on the mode of action of amantadine. Acta Pharmacol. Toxicol..

[21] Peeters M., Romieu P., Maurice T., Su T.P., Maloteaux J.M., Hermans E. (2004). Involvement of the sigma 1 receptor in the modulation of dopaminergic transmission by amantadine. Eur. J. Neurosci..

[22] Otton H.J., McLean A.L., Pannozzo M.A., Davies C.H., Wyllie D.J.A. (2011). Quantification of the Mg2+-induced potency shift of amantadine and memantine voltage-dependent block in human recombinant GluN1/GluN2A NMDARs. Neuropharmacology.

[23] Krzystanek M., Pałasz A. (2020). Possibility of a New Indication for Amantadine in the Treatment of Bipolar Depression—Case Series Study. Pharmaceuticals.

[24] Lemmer B. (1973). Effects of amantadine and amphetamine on serotonin uptake and release by human blood platelets. Eur. J. Pharmacol..

[25] Welsh J.P., Placantonakis D.G., Warsetsky S.I., Marquez R.G., Bernstein L., Aicher S.A. (2002). The serotonin hypothesis of myoclonus from the perspective of neuronal rhythmicity. Adv. Neurol..

[26] Rissardo J.P., Caprara A.L., Durante Í., Rauber A. (2022). Lithium-associated movement disorder: A literature review. Brain Circ..

[27] Rissardo J.P., Caprara A.L.F. (2023). Fluoroquinolone-Associated Movement Disorder: A Literature Review. Medicines.

[28] Kim H.S., Park I.S., Lim H.K., Choi H.S., Oh S., Park W.K., Jang C.G., Kim S.H., Chang M.J. (2000). N-Methyl-D-aspartate receptor antagonists enhance the head-twitch response, a 5-hydroxytryptamine2 receptor-mediated behaviour, in reserpine-treated mice. J. Pharm. Pharmacol..

[29] Simon D.K., Tanner C.M., Brundin P. (2020). Parkinson Disease Epidemiology, Pathology, Genetics, and Pathophysiology. Clin. Geriatr. Med..

[30] Pena A.B., Caviness J.N. (2020). Physiology-Based Treatment of Myoclonus. Neurotherapeutics.

[31] Horadam V.W., Sharp J.G., Smilack J.D., McAnalley B.H., Garriott J.C., Stephens M.K., Prati R.C., Brater D.C. (1981). Pharmacokinetics of amantadine hydrochloride in subjects with normal and impaired renal function. Ann. Intern. Med..

[32] Hayden F.G., Minocha A., Spyker D.A., Hoffman H.E. (1985). Comparative single-dose pharmacokinetics of amantadine hydrochloride and rimantadine hydrochloride in young and elderly adults. Antimicrob. Agents Chemother..

[33] Liu P., Cheng P.J., Ing T.S., Daugirdas J.T., Jeevanandhan R., Soung L.S., Galinis S. (1984). In vitro binding of amantadine to plasma proteins. Clin. Neuropharmacol..

[34] Brenner M., Haass A., Jacobi P., Schimrigk K. (1989). Amantadine sulphate in treating Parkinson’s disease: Clinical effects, psychometric tests and serum concentrations. J. Neurol..

[35] Köppel C., Tenczer J. (1985). A revision of the metabolic disposition of amantadine. Biomed. Mass. Spectrom..

[36] Jiménez-Jiménez F.J., Puertas I., Toledo-Heras M. (2004). Drug-induced myoclonus: Frequency, mechanisms and management. CNS Drugs.

[37] Degelau J., Somani S., Cooper S.L., Irvine P.W. (1990). Occurrence of adverse effects and high amantadine concentrations with influenza prophylaxis in the nursing home. J. Am. Geriatr. Soc..

[38] Factor S.A., Molho E.S., Brown D.L. (1998). Acute delirium after withdrawal of amantadine in Parkinson’s disease. Neurology.

[39] Oertel W., Eggert K., Pahwa R., Tanner C.M., Hauser R.A., Trenkwalder C., Ehret R., Azulay J.P., Isaacson S., Felt L. (2017). Randomized, placebo-controlled trial of ADS-5102 (amantadine) extended-release capsules for levodopa-induced dyskinesia in Parkinson’s disease (EASE LID 3). Mov. Disord..

