# Supplementary material for: Myoclonus Secondary to Amantadine: Case Report and Literature Review
Source: Clin Pract. 2023 Jul 20;13(4):830–7. doi: 10.3390/clinpract13040075 (PMC10366862; doi:10.3390/clinpract13040075)
Supplement: Supplementary file 1 [file clinpract-13-00075-s001.zip › Table S1 - FreeText.pdf]

**Supplemental Material – FreeText and MeSH search terms in the US National Library of Medicine**		
Category	Search terms	
	Amantadine	1-Adamantylamine
Myoclonus	("amantadin"[All Fields] OR "amantadine"[MeSH Terms] OR "amantadine"[All Fields] OR "amantadines"[All Fields]) AND ("myoclonus"[MeSH Terms] OR "myoclonus"[All Fields])	("amantadine"[MeSH Terms] OR "amantadine"[All Fields] OR "1 adamantylamine"[All Fields]) AND ("myoclonus"[MeSH Terms] OR "myoclonus"[All Fields])
Movement disorder	("amantadin"[All Fields] OR "amantadine"[MeSH Terms] OR "amantadine"[All Fields] OR "amantadines"[All Fields]) AND ("movement disorders"[MeSH Terms] OR ("movement"[All Fields] AND "disorders"[All Fields]) OR "movement disorders"[All Fields] OR ("movement"[All Fields] AND "disorder"[All Fields]) OR "movement disorder"[All Fields])	("amantadine"[MeSH Terms] OR "amantadine"[All Fields] OR "1 adamantylamine"[All Fields]) AND ("movement disorders"[MeSH Terms] OR ("movement"[All Fields] AND "disorders"[All Fields]) OR "movement disorders"[All Fields] OR ("movement"[All Fields] AND "disorder"[All Fields]) OR "movement disorder"[All Fields])
